# 

**Published:** 1892-11-26

**Authors:** 


**Notices of Births, Deaths, and Marriages,** are inserted in this column at a charge of 2s. 6d. for an announcement not exceeding four lines.

### NOTICE TO CORRESPONDENTS.

All MS., Letters, Books for Review, and other matters intended for the Editor should be addressed The Editor, The Lodge, Porchester Square, London, W.

The Editor cannot undertake to return rejected M.S., even when accompanied by stamped directed envelope.

**Notice to Advertisers and Subscribers.**

All Advertisements, Orders for Copies of The Hospital, and Business Communications should be addressed to The Publisher, *not to the Editor*, The Hospital, 140, Strand, W.C.

The Cost of Subscription, post free, is as follows:—Per week, 2½d.; per quarter, 2s. 6d.; per half-year, 5s.; per annum, 8s. 6d. Foreign:—Per annum, 10s. 8d.; payable, in all cases, in advance.

**"Burdett's Hospital Annual," 1892—1893.**

Fourth year of publication. 700 pp. Boards, gilt lettering. A most complete guide to British and Colonial Hospitals, Dispensaries, Nursing and Convalescent Institutions, and Asylums. Price 5s. Published by The Scientific Press (Limited), 140, Strand, W.C.

**NOTICE.—The Index for Vol. XII. (ending September 1892) is on sale at the Office of this Journal, price 2½d., post free.**

### BOOKS RECEIVED.

SMITH, ELDER, AND CO.

"Croonian Lectures for 1892." By Sir W. Roberts, M.D.

C. GRIFFIN AND CO.

"A Manual of Inorganic Chemistry." By A. Dupré, Ph.D., and H Wilson Hake, Ph.D.

KEGAN PAUL, TRENCH, AND TRUBNER.

"First Aid in Illness and Injury." By James E. Pilcher, M.D.

CAMBRIDGE UNIVERSITY PRESS.

"Atlas of Clinical Medicine," Vol. II., Part I. By Byrom Bramwell, M.D.

SWAN SONNENSCHEIN AND CO.

"Geographical Distribution of Disease in Great Britain." By Alfred Haviland, M.R.C.S.

"Introduction to the Study of Elementary Biology." By H. J. Campbell, M.D.

"The Social Horizon." By the author of "Life in Our Villages."

"Introduction to Physiological Psychology." By Dr. T. Ziehnen.

"Text-book of the Embryology of Man and Mammals." By Dr. Oscar Hertwig; translated by Edward Mark, Ph.D.

**Periodicals and Pamphlets.**

RELIGIOUS TRACT SOCIETY.—The Sunday at Home, The Girl's Own Paper, The Boy's Own Paper, The Leisure Hour, Friendly Greetings.

**Other Publications.**

Advice to Women on the Care of their Health during and after Confinement, by Florence Stacpoole; Our Sick and How to Take Care of Them, or Plain Teaching on Sick Nursing at Home, by Florence Stacpoole; The Medical Magazine; The Rural World; The Record of Technical and Secondary Education, for November; The Therapist; The Clinical Journal; Report of the Medical and Surgical Cases at the London Temperance Hospital for three years; The Vaccination Inquirer; St. Thomas's and Guy's Hospital Gazettes; The Review of Reviews; The Practical Side of Scientific Temperance.

### SCRAPS AND GLEANINGS.

The Hospital Saturday street collections in Cork amounted this year to £349 0s. 11¼d.

Mrs. Brown, a benevolent lady who died at Adelaide a short time ago, has bequeathed £100 000 to the Home for Crippled Children, and to the Convalescent Home for the Infant Poor.

In consequence of the small-pox epidemic at Warrington, football matches have been cancelled for last Saturday next between Warrington v. Salford and Warrington with Widnes.

It is proposed to hold a Chemists and Druggists' Exhibition at the Agricultural Hall in the spring of next year. The dates as at present fixed are from the 22nd to the 30th of April, 1893.

An entertainment was given on Tuesday, the 14th inst., to the patients at Brompton Hospital. Miss Edie Reynolds gave several violin solos, and other friends contributed songs and pianoforte solos.

After deducting expenses in connection with the Brighton Hospital Saturday Fund, a balance of £1,22[illegible] 16s. 11¼d. was left for distribution amongst the various medical institutions connected with the town.

There were 552 patients admitted to the Ely Dispensary during the past twelve months. In addition 300 were visited in their own homes, and 92 remained under treatment. The total income for the year amounted to £242 11s. 6d.

It is stated that a Royal Commission is shortly to be appointed to consider whether any alterations in the Poor Law are desirable with regard to the cases of persons whose destitution is caused by incapacity for work resulting from old age.

The late Mr. William Hodgson, of Haxby, near York, has bequeathed a legacy of £1,000 to the York County Hospital, £1,000 to the Wilberforce School for the Blind, at York, and £500 to the York County Asylum, besides legacies to other institutions.

At the monthly meeting of the Governors of the Derry City and County Infirmary the secretary announced that the late Mr. Robert Adair, of Brisbane, had left a sum of £1,000 to found a ward in the Derry Infirmary, to be called the Adair Ward.

The Duke of York will next month open the Royal Eye Hospital, Southwark. The foundation stone was laid by the Prince of Wales in July, 1890. The institution, which was founded in 1857, has latterly been relieving upwards of 7,000 patients annually.

A donation of £46 1s., the proceeds of a collection at Harrow School on Sunday, November 13th, after a special sermon by the Rev. C. J. Ridgeway, Vicar of Christ Church, Lancaster Gate, has been received at the Seaside Camp for London Working Boys, Northumberland Chambers.

Most satisfactory reports have been issued showing the work done in the Northallerton Cottage Hospital, and also the Fever Hospital. Two cases of small-pox and several cases of typhoid fever have been treated in the latter institution during the year, besides four patients admitted to the Cottage Hospital.

Census of metropolitan paupers, taken on the last day of the fifth week of October, 1892 (exclusive of lunatics in asylums and vagrants), was indoor, 60,144; outdoor, 32,779; total, 92 923. Vagrants relieved in the metropolis on the same date, men, 777; women, 203 children under 16, 20; total, 1,000.

From December 26th the use of intoxicants amongst the patients and attendants of the Essex County Asylum are to be discontinued. The patients, it is reported, are to have milk or lime-juice instead of beer, &c., and the under officials are to receive an addition of from £2 to £5 per annum to their wages.

The Doncaster Dispensary has now been established 100 years. The annual report of the infirmary just published shows the good work which has been done by the united institutions. During the past year the total number of cases admitted to the infirmary have been 174, and of dispensary patients 2,439.

The debt of £500 which for the past few years has been upon the Alexandra Hospital, Woodhall Spa, has now been paid off, but much still remains to be completed in the hospital, and in order to start clear of debt in the spring, the committee have decided to close the building during the winter months.

At the opening of the new central offices of the Belfast Dispensary, Mr. Browne, the chairman, and Mr. Hamil, the vice-chairman, invited all the guests to a champagne luncheon. The new building is situated in Glengal Street, and is well planned and constructed, and fitted up with all modern improvements.

The sad death of Dr. Gilpin from an attack of typhoid fever, contracted in the discharge of his duties, is reported. Dr. Gilpin was only 27 years of age, and for the past two years had held the appointment of Medical Officer of Health to the Stratford-on-Avon Corporation, and was also Medical Officer to the Rural Sanitary Authority.

The Liverpool Hospital Sunday Fund for this year amounted to only £6,410, showing a decrease of £563, which was collected in 1891. This is a very serious loss on the principal loss of revenue, but the committee trust that another year will prove that the decrease is to be ascribed to temporary causes rather than to a waning interest in their great charities.

The Duke of Westminster has forwarded £100 to the Central Committee for the relief of the distress in East London. Many others have also intimated their willingness to forward the work. The committee estimate that £8 will be sufficient to aid one family to tide over the winter, and anyone wishing to help should communicate with Mr. Peer, Secretary, Clearing House for Unemployed, Polytechnic, Regent Street.

The Mayor of Manchester, accompanied by the Mayoress, formally opened, on the 7th inst., a temporary cancer pavilion and home which is intended to form a branch of a large general hospital, or one of a group of hospitals, connected with Owens College. A large house has been secured on the estate in Stanley Grove, handed over to the Owens College authorities by the Whitworth trustees, and benefactions from the Proctor trustees, Mr. Chancellor Christie and others, have enabled the committee to provide some 17 beds for cancer patients.

### THE STUDENT OF MEDICINE.

[All communications for this Supplement should be addressed to The Editor of The Hospital, at 140, Strand, with the words, "Students' Column," written in the left hand top corner of the envelope.]

### NOTES FROM THE SCHOOLS.

### Anderson College Medico-Chirurgical Society, Glasgow.

President—A. M. Buchannan, M.A., M.D., F.F.P.S.G.

The second meeting of this society was held in the college buildings on Saturday, the 12th inst., when a large gathering took place, it being the occasion of the honorary president's inaugural address. Professor A. M. Buchannan, M.A., M.D., &c., took for his subject, "Medical Science, its Uncertainties, and How to Overcome Them." In treating of his subject, he urged upon his audience the importance of close attention to facts, their proper association and correct generalisation. At the close a hearty vote of thanks was accorded to him, and he received quite an ovation, the members cheering loudly.

### APPOINTMENTS.

Aldous, G. F., L.R.C.P.Lond., M.R.C.S., re-appointed Medical Officer to the Plymouth Public Dispensary. Ashe, E. Oliver, M.D.Lond., F.R.C.S.Eng., appointed Senior House-Surgeon to the Kimberley Hospital, South Africa. Body, H. M., M.R.C.S.Eng., re-appointed Medical Officer of Health for Crediton Urban Sanitary District. Buchan, W. A., M.B., C.M.Edin., re-appointed Medical Officer to the Plymouth Public Dispensary. Clark, H.E., M.R.C.S., appointed Professor of Systematic Surgery. Duke, Allen Forrester, L.R.C.P.Edin., M.R.C.S.Eng., appointed Medical Officer for the Second District of the Cheltenham Union. Ellison, J., M.D., M.Ch.Irel., appointed Health Officer for the Shire of Numurkah, Victoria, Australia. Fordyce, B. E., M.B., C.M.Edin., appointed Medical Officer of the Sixth Sanitary District; also to the Workhouse; and Vaccination Officer of the Chesterton Union. Fox, E. L., M.D., B.Ch.Camb., M.R.C.P.Lond., M.R.C.S., appointed Second Physician to the Plymouth Public Dispensary. Frost, John Kingdon, M.R.C.S.Eng., appointed Medical Officer for the Madley District of the Abbeydore Union. Gemmell, Samson, M.D., C.M., F.F.P.S.Glas., appointed Visiting Physician to the Western Infirmary, Glasgow. Gibbs, S. A., M.B., C.M.Edin., appointed Honorary Surgeon to the H Battery New Zealand Regiment Artillery Volunteers. Gornall, J. G., M.A., M.B.Cantab., M.R.C.S., L.R.C.P., appointed Assistant Medical Officer of Health for the Borough of Warrington, and Medical Superintendent of the Hope Hospital for Small-pox. Harris, A. W., M.R.C.S., D.P.H.Eng., appointed Port Sanitary Medical Officer for Southampton. Hillier, T. Ernest, M.A., M.B.Cantab., M.R.C.S., L.S.A., appointed Medical Superintendent to the Infirmary and Medical Officer of the Workhouse of the parish of Paddington. Hodson, Frederick, M.R.C.S.Eng., L.S.A., appointed Medical Officer of the Hornsea-with-Hornsea Burton Urban Sanitary District of the Skirlaugh Union. Hogg, G. H., M.B., C.M.Edin., appointed Assistant House Surgeon to Clayton Hospital, Wakefield. Hooper, Alfred, M.R.C.S., re-appointed Medical Officer of Health for the Second Sanitary District of the Burton-on-Trent Union. Jamie, R. W., M.B., C.M.Edin., D.P.H.Camb., appointed Medical Officer of Health for the Coalville Urban Sanitary District of the Ashby-de-la-Zouch Union. Johnston, J., M.R.C.S.Eng., appointed Surgeon to the Maidstone Police. Johnston, T. L., L.R.C.P., L.R.C.S., L.F.P.S.G. and L.M., appointed Second Assistant Medical Officer Berkshire County Asylum. Knox, David N., M.A., M.B., appointed Professor of Clinical Surgery to St. Mungo's College, Glasgow. Lempriere, C.L., M.B., C.M.Edin., appointed Honorary Medical Officer of the Melbourne Hospital for Sick Children, Victoria, Australia. Marsden, Richard Walter, M.R.C.S.Eng., L.R.C.P.Lond., appointed Assistant Medical Officer of the Workhouse and Receiving Wards of the Township of Manchester. May, William Page, M.D., B.Sc.Lond., M.R.C.S., M.R.C.P., appointed Pathologist to the City of London Hospital for Diseases of the Chest, Victoria Park. Monckton, William, L.R.C.P.Edin., M.R.C.S.Eng., appointed Medical Officer to the Portishead Local Board. Osburn, H. B., M.R.C.S., L.R.C.P., D.P.H.Camb., appointed Medical Officer to the Parish of Bagshot, Surrey. Paine, William Henry, L.R.C.P.Lond., M.R.C.S. Eng., appointed Medical Officer of the West Green District of the Edmonton Union. Porter, C., M.D., B.Ch., B.A.O.Roy. Univ.Irel., D.P.H.Camb., M.R.C.S.Eng., appointed Medical Officer of Health, Physician to Fever Hospital, and Police Surgeon for County Borough of Stockport.

### THE STUDENT OF MEDICINE—(*continued*).

### VACANCIES.

#### Resident Medical Officers.

Brighton, Hove, and Preston Dispensary, House Surgeon to the Western Branch, unmarried, doubly qualified—Salary £140 per annum (less board at 8s. per week), with furnished apartments, coal, gas, and attendance. Applications to the Assistant Secretary by December 15th.

City of London Hospital for Diseases of the Chest, Victoria Park, E., House Physician—Board, residence, and allowance for washing provided. Appointment for six months. Applications to the Secretary at the Office, 24 Finsbury Circus E.C., by December 8th.

Devon and Exeter Hospital, Exeter, Assistant House Surgeon, doubly qualified, married—Salary £40 per annum, with board and lodging. Applications to G. A. Townsend, Secretary, by November 26th.

General Hospital, Barbadoes, West Indies, Junior House Surgeon—Salary £200 per annum, and quarters. Appointment for three years. Applications to the Secretary by December 1st.

General Hospital for Sick Children, Pendlebury, Manchester, Junior Resident Medical Officer, doubly qualified—Salary, £80 per annum. Applications to the Chairman of the Medical Board by November 30th.

Great Western Dispensary, Marylebone Road, N.W., Junior House Surgeon, unmarried—Salary £50 per annum, with board and apartments. Applications to the Honorary Secretary.

Monkstown Hospital, County Dublin, Resident Medical Officer—Salary £40 per annum, with board, apartments, gas, and coal. Applications to the Honorary Secretary by November 14th. Election on December 1st.

Noble's Isle of Man General Hospital and Dispensary, Athol Street, Douglas, Isle of Man. Resident House Surgeon, doubly qualified, unmarried—Salary, £100 per annum, with apartments, gas, coal, and laundry free. Applications to F. Brown, Honorary Secretary, by December 3rd.

North-Eastern Hospital for Children, Hackney Road, N.E., Junior House Surgeon, doubly qualified—Salary £60 per annum. Applications to Alfred Nixon, Secretary, 27, Clement's Lane, E.C., by December 9th.

Northern Infirmary, Inverness, House-Surgeon and Dispenser—Salary £75 per annum, with board, &c. Applications to the Honorary Secretary, Mr. Duncan Shaw, W.S., by November 26th.

Royal Infirmary of Edinburgh, Superintendent, must be member of medical profession—Salary £500 per annum, with free house and gas. Applications to Mr. James S. Trainer, Treasurer and Clerk, by December 5th.

Royal Westminster Ophthalmic Hospital, House Surgeon—Must be legally qualified and possess some knowledge of ophthalmic surgery; board and residence. Applications, with testimonials, on or before November 30th inst. To take office January 1st, 1893.

West London Hospital, Hammersmith Road, W., House Physician. Appointment for six months. Board and lodging provided. Applications to R. J. Gilbert, Secretary, by December 7th.

West London Hospital, Hammersmith Road, W., House Surgeon. Appointment for six months. Board and lodging provided. Applications to R. J. Gilbert, Secretary, by December 7th.

#### Non-Resident Medical Officers.

Enniscorthy Union, Medical Officer to Oulart Dispensary—Salary £125 per annum, and fees. Applications to Mr. Laurence Kelly, Honorary Secretary, Castle Ellis, Ballaghkeene. Election on November 29th.

Enniskillen Union, Medical Officer to Holywell Dispensary—Salary £135 per annum, and fees. Applications to Mr. Phibbs Nixon, Honorary Secretary, Thornhill House, Gortahill. Election on November 26th.

London Throat Hospital, 204, Great Portland Street, W., Pathologist. Applications to Edward Low, Secretary to the Medical Committee, by December 2nd.

Metropolitan Hospital, Kingsland Road, N.E., Assistant Physician. Applications to Charles H. Byers, Secretary, by November 28th.

Royal College of Surgeons of England.—Election to the Court of Examiners. Applications to the Secretary by November 30th.

St. Mungo's College, Glasgow, Chair of Anatomy. Applications to Henry Lamond, Secretary, by December 1st.

Strand District of the City of London, Medical Officer of Health, not more than 45 years of age. Salary £400 per annum. Applications marked outside "Medical Officer's Appointment," to be sent to the Offices of the Board, 5, Tavistock Street, Strand, W.C., by November 26th.

Victoria Hospital, Burnley, Honorary Ophthalmic and Aural Surgeon. Applications, endorsed "Ophthalmic," to the Honorary Secretary, Joshua Rawlinson, J.P., by December 6th.

